# Hospital Meetings

**Published:** 1896-02-29

**Authors:** 


### HOSPITAL MEETINGS.

### ST. THOMAS'S HOSPITAL.

Opening of the Beatrice and Florence Wards by the Duke of Connaught.

The opening of two of the five hitherto closed wards on Friday in last week marked a red-letter day in the annals of St. Thomas's Hospital, and all the arrangements for the ceremony were worthy of the occasion. The great Central Hall, where in 1871 the Queen herself declared the newly-built hospital open, was full to overflowing long before the hour fixed for the arrival of the Duke and Duchess of Connaught, the audience numbering many faces familiar in the hospital world. The Archbishop of Canterbury was present, also the Bishop of Rochester, the Lord Mayor and Lady Mayoress, Sir J. Stuart Knill, Sir Henry Doulton, Sir William MacCormac, and many others. The Duke and Duchess of Connaught arrived soon after half-past three, and were received at the main entrance in Lambeth Palace Road by Mr. Wainwright, the treasurer, and the hospital authorities, by whom they were conducted to the platform erected at the far end of the hall.

The address of welcome, read by Mr. Wainwright, stated that after the enthusiasm which was aroused by the opening of the hospital had subsided unexpected demands and other causes made it impossible to keep all the wards open, and five of them had to be closed. At the same time the population of South London was increasing rapidly, and at present the district which the hospital helped to serve numbered some 1,500,000 people, the hospital accommodation being only 926 beds. Explaining the circumstances which warranted the reopening of the two wards, the Treasurer reminded the Duke of Connaught that in his capacity as President his Royal Highness had appealed for public support a year ago, and that in furtherance of that appeal a meeting was held at the Mansion House, when £100,000 was asked for. It had been hoped that a larger response would have been made, but circumstances had been unfavourable, and only £27,000 had been received. For some years past about £6,000 per annum had been devoted towards the liquidation of the debt incurred in rebuilding the hospital, and the committee had now repaid half of the remaining debt, and a sum of about £5,000 would be available for the purposes of the hospital. It was now intended to open two wards, accommodating 30 patients each, which would provide for the treatment of some 900 extra in-patients yearly. He hoped that they would be able to keep the wards open, but in order to do so it would be necessary that they should appeal for annual subscriptions. In the two wards to be re-opened alterations had been made so as to continue the traditions that had placed St. Thomas's Hospital in the forefront of English hospitals for the successful treatment of disease, and to ensure the efficiency of the nursing school which was originally organised by Miss Florence Nightingale, and in the welfare of which she still took a most active interest.

A letter had been received from Miss Nightingale enclosing a cheque for £100.

The Duke of Connaught, replying to the address, said: My Lord Archbishop, my Lord Mayor, ladies and gentlemen, I am now asked to declare the two wards, named Beatrice and Florence, to be open. It is a great satisfaction to me that the endeavours of the hospital authorities to reopen some of the wards which had been closed for some years have met with success. Thanks to the Lord Mayor, who rendered so much help by the meeting which was held at the Mansion House last year, we have obtained our present success. It is of course a great disappointment that we have only got £27,000 instead of £100,000; but I venture to think that half a loaf is better than none at all, and we must be very thankful that we have met with so large a response as we have. There are great difficulties in the way of providing funds for this and similar other institutions of which we are in this kingdom so proud. I cannot help thinking that this reopening to-day will prove to the public that the hospital exists for a really practical purpose. There is a very peculiar difference between North and South London in the matter of hospital accommodation which is very striking. In North London with a population of about 2,500,000, they have twelve or fourteen hospitals, with accommodation for 7,000 beds; while in South London, with a population of 1,500,000, there are but two hospitals and accommodation for 926 beds. Surely, such facts as these should encourage others to come to the assistance of South London, which, as we all know, has a teeming population mostly of the poorest class. They are unable to subscribe as their richer brethren do, and I hope those who are able will remember their poorer brothers. I will not refer at length to the history of this ancient and noble institution. It was founded in the reign of Edward VI., and enjoyed the support of that Sovereign and the City of London, and we are very grateful to the City for the warm interest they have always taken in the hospital, and which, I am sure, they will always continue. I have now great pleasure in declaring these wards open.

After a short service of dedication, conducted by the Archbishop of Canterbury, the Duke and Duchess of Connaught inspected the two wards, in each of which a hymn was sung by the choir of the Royal Military Chapel, Wellington Barracks.

The visitors found tea ready for them in the long corridor, and afterwards visited the wards and the new nurses' home, into which the apothecaries' house has been converted. The newly-opened wards were a pleasure to see, fitted as they are with every appliance for the comfort of the patients. The electric light has been laid on in these wards as well as in the new nurses' quarters.

### CHARING CROSS HOSPITAL.

Annual General Court.

The annual court of the governors of Charing Cross Hospital was held in the board-room on Wednesday afternoon, Mr. John B. Martin (treasurer) taking the chair. There was a good attendance of governors, though no one seemed to have much to say upon the points in the report commented on by the chairman, contenting themselves for the most part with a more or less formal proposal of the various resolutions on the agenda. Mr. Martin, in moving the adoption of the report, alluded to the loss the hospital had sustained in the death of Mr. Callum, and then went on to speak of the financial position of the institution, which was, undoubtedly, a very anxious one. Two important questions had come before the council for consideration. It had been suggested that the hospital should be removed to Camberwell, but this had been emphatically negatived by the council, a course in which he thoroughly concurred. The fact that the number of patients treated at the hospital continued to increase went to prove that it was needed in its present position. Then the lease of Toole's Theatre had expired during the year, and as its renewal would have necessitated an outlay of £4,000 to £5,000, and as, moreover, the proximity of the theatre was a source of anxiety on account of risk of fire, and also interfered with the access of light and air to the hospital, it had been decided not to re-let the building again as a theatre, in spite of the annual loss of £1,000 suffered in consequence. The new convalescent home would be opened in April or May of the present year, and H.R.H. the Prince of Wales had been asked to perform the opening ceremony, and had promised to consider the invitation in arranging his summer engagements. Reverting again to the financial condition of the hospital, Mr. Martin remarked that the legacies (amounting to £2,468) had fallen off, showing a decrease of £546 as compared with the previous year, and he attributed this in a certain degree to a hesitation on the part of some people to leave money to individual hospitals which might in the event of the establishment of a Central Hospital Board become diverted from its original purpose. The report showed that the number of patients treated during 1895 amounted to 24,949, of whom 2,117 were in-patients, 11,552 were out-patients, and 11,280 casualties. The ordinary income showed a decrease as compared with the previous year, but not so marked a difference as usual between a festival year (1894) and its successor. The ordinary expenditure chargeable to the general account amounted to £14,394 19s. 5d. The council anticipated that an additional sum of £3,000 would be required over or above the ordinary amount for the coming year, of which £2,000 would be wanted to meet the expenses of the Convalescent Home, and £1,000 to compensate for the loss on Toole's Theatre. The report was unanimously adopted. The Rev. J. F. Kitto, in proposing a vote of thanks to the chairman, remarked that the income of the hospital appeared to be annually £4,000 less than its expenditure, a cavern which contracting large loans would not bridge over. He thought this accumulating deficit ought to be arrested, and that the council and the treasurers would not, and ought not to, be comfortable till that object had been attained. It would, he said, perhaps have been better for Charing Cross Hospital if they had a stern banker who would not allow a large overdraft, which would make some tremendous effort absolutely imperative.

### HOSTEL OF ST. LUKE.

The third annual meeting of the Hostel of St. Luke was held recently at the Church House, when the chair was taken by the Bishop of Marlborough. A good many clergy and laity interested in the society were present. The Hostel, it will be remembered, was established for the benefit and help in time of sickness of the poorer clergy and their families. The Chairman, after the proceedings had been opened with prayer, said the Hostel of St. Luke was doing a good, a blessed, and a Christian work, and no one in all England knew better than he the benefits which this society had been giving to the poorer clergy and their families. There was no society more thoroughly deserving of the support of Christian people than the Hostel of St. Luke, and, in conclusion, he urged those who were present to do more in the future than had been done in the past. Lord Ashcombe moved the adoption of the report, and spoke of the increased usefulness of the Hostel and of their indebtedness to those medical men who gratuitously gave their services. Their indebtedness, he ventured to think, was much greater than was fully realised. Canon Phillips seconded the resolution, which was carried unanimously. The Bishop of Truro proposed the following resolution: "That Clause 2 of the constitution be altered to read as follows: 'To provide medical and surgical treatment and a nursing home for the clergy of the Anglican Communion, their wives, widows, and children.'" Dr. Champneys seconded the resolution, which was supported by Canon Barker, and carried.

### ST. JOHN'S HOSPITAL FOR SKIN DISEASES.

The Earl of Chesterfield presided at the thirty-second annual meeting of this hospital, held at the Hotel Victoria on the 21st inst. In moving the adoption of the report, Lord Chesterfield remarked that it gave him great satisfaction to be able to say that the year 1895 had been one of continued prosperity and progress. His ambition for the welfare of the hospital knew no bounds, and he felt it was at least satisfactory, considering that they heard on all sides of hospitals getting into debt, to be able to say that the prospects of St. John's Hospital were of the best and cheeriest. For the first time for twenty years it was out of debt. Some people might say it was a bad thing for a hospital to be out of debt, but for himself he preferred to be the president of a solvent institution. Now, however, there would be increased expenditure to face, and they must put their shoulders to the wheel and work hard to maintain the present position of the hospital. The report stated that the receipts for the year had amounted to £3,806 13s. 4d., as compared with £3,645 1s. 1d. in the previous year; the expenditure had been £3,435 1s. 11d., a decrease of £61 compared with last year. The new hospital in the Uxbridge Road provided 40 beds, and for the expenses of fitting an earnest appeal must be made for special contributions.

### QUEEN CHARLOTTE'S LYING-IN HOSPITAL.

The annual meeting of Queen Charlotte's Hospital was held on Monday last, February 24th, Dr. G. Bernard Brodie presiding. The committee reported that more patients had been relieved during 1895 than in any previous year since the hospital was founded in 1752, 1,124 patients being treated as inpatients, and 1,370 at their own homes. The income amounted to £4,530 0s. 11d., the expenditure was £5,001 6s. 2d., giving a deficit of £561 5s. 3d. on the year's working. Additional support is also much needed for the convalescent home at Kilburn. The committee are negotiating for a site on which to build a nurses' home, and hope soon to be able to report that it has been acquired. Certain necessary alterations and improvements to the hospital itself have also been decided upon.

